# Fatal Avian Influenza A H5N1 in a Dog

**DOI:** 10.3201/eid1211.060542

**Published:** 2006-11

**Authors:** Thaweesak Songserm, Alongkorn Amonsin, Rungroj Jam-on, Namdee Sae-Heng, Nuananong Pariyothorn, Sunchai Payungporn, Apiradee Theamboonlers, Salin Chutinimitkul, Roongroje Thanawongnuwech, Yong Poovorawan

**Affiliations:** * Kasetsart University, Nakorn Pathom, Thailand;; † Chulalongkorn University, Bangkok, Thailand

**Keywords:** H5N1, Dog, Influenza A, Thailand, research

## Abstract

Avian influenza H5N1 virus is known to cross the species barrier and infect humans and felines. We report a fatal H5N1 infection in a dog following ingestion of an H5N1-infected duck during an outbreak in Thailand in 2004. With new reports of H5N1 virus continuing across Asia, Europe, and Africa, this finding highlights the need for monitoring of domestic animals during outbreaks.

Highly pathogenic avian influenza (HPAI) H5N1 has spread across Asia, Europe, and Africa. Not limited to poultry, the virus has also been shown to cross the species barrier infecting humans (*1*) and felines, including domestic cats (*2*) and tigers (*3**–**5*). Both cats and tigers were reported as becoming infected after eating poultry carcasses harboring HPAI. Here, we report a case of HPAI H5N1 infection in a domestic dog following ingestion of the carcass of an infected duck.

## The Study

In October 2004, the carcass of an a ≈1-year-old dog from Suphanburi Province, Thailand, was submitted for necropsy at the Faculty of Veterinary Medicine, Kasetsart University, in Nakorn Pathom, Thailand. The dog's owner stated that the dog had eaten duck carcasses from an area with reported HPAI H5N1 infections in ducks. Approximately 5 days after ingesting the carcasses, the dog developed high fever, panting, and lethargy and died on the following day. Within 4 hours of its discovery, the dog carcass was sent to the laboratory.

**Figure 1:**
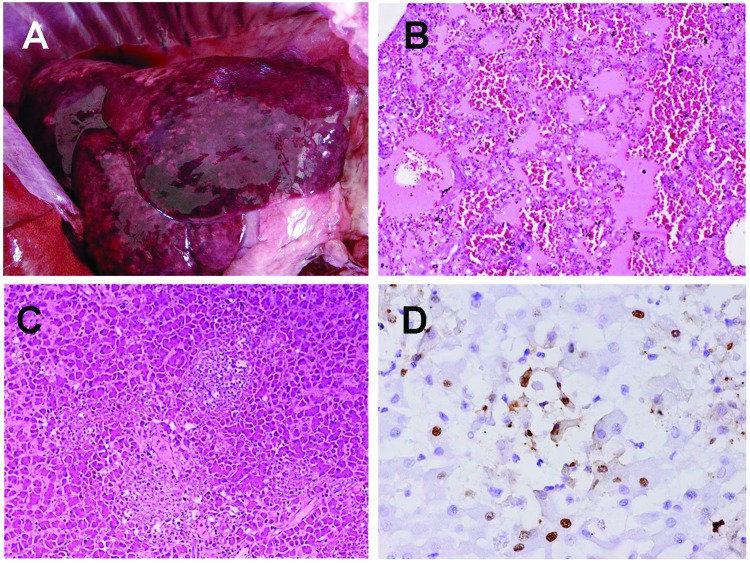
Gross and microscopic lesions from dog infected with highly pathogenic avian influenza (HPAI) H5N1. A) Severe congestion and edema in the lung. B) Lung histopathologic results showing severe pulmonary edema and hemorrhage with black-brown particles (hemosiderin) (magnification ×100). C) Liver histopathologic changes showing necrotic foci (pale area) (magnification ×100). D) Immunohistochemical results: the nucleoprotein of the virus is detected in nuclei of hepatocytes with brown granule (magnification ×200).

Necropsy findings included bloody nasal discharge; severe pulmonary congestion and edema (Figure 1A); and congestion of the spleen, kidney, and liver. Brain, lung, trachea, heart, duodenum, jejunum, ileum, liver, spleen, kidney, pancreas, and urine specimens were obtained separately and processed for virus isolation by injection into 10-day-old embryonated chicken eggs. Forty-eight hours later, allantoic fluids harvested from dead embryos that had been injected with supernatants of ground brain, trachea, lung, intestine, liver, and kidney were tested with the hemagglutination and hemagglutination-inhibition tests. Influenza virus was isolated from lung, liver, kidney, and urine specimens, and the viral subtype was determined to be H5N1 by reverse transcription (RT)–PCR (*6*). The 4 tissues that showed virus were also processed for histopathologic and immunohistochemical analysis. Immunohistochemical tests were performed on paraffin-embedded tissues by using a mouse monoclonal antibody anti-nucleoprotein of influenza A H5N1 (B.V. European Veterinary Laboratory, Woerden, the Netherlands) as a primary antibody and a polyclonal goat antimouse immunoglobulin G tagged with peroxidase as a secondary antibody (DAKO A/S, Glostrup, Denmark). Diamino-benzidine was used as a substrate. Positive lung tissue from the dog that was incubated with phosphate-buffered saline instead of the mouse monoclonal antibody antinucleoprotein of influenza A H5N1, and tissue from the liver and lung of a cat killed by a car served as negative control (*2*).

Histopathologic examination of the lung showed severe pulmonary edema and interstitial pneumonia with inflammatory cell infiltration. Hemolysis with brownish black particles was found in the pulmonary parenchyma (Figure 1B), and the liver showed focal necrosis (Figure 1C). The kidneys showed mild nephritis with tubular degeneration. No microscopic lesions were found in any other organs. On immunohistochemical analysis, positive sites were found in alveolar cells, hepatic cells (Figure 1D), renal tubular epithelium, and glomerulus; none of the remaining organs were positive for H5N1.

**Figure 2:**
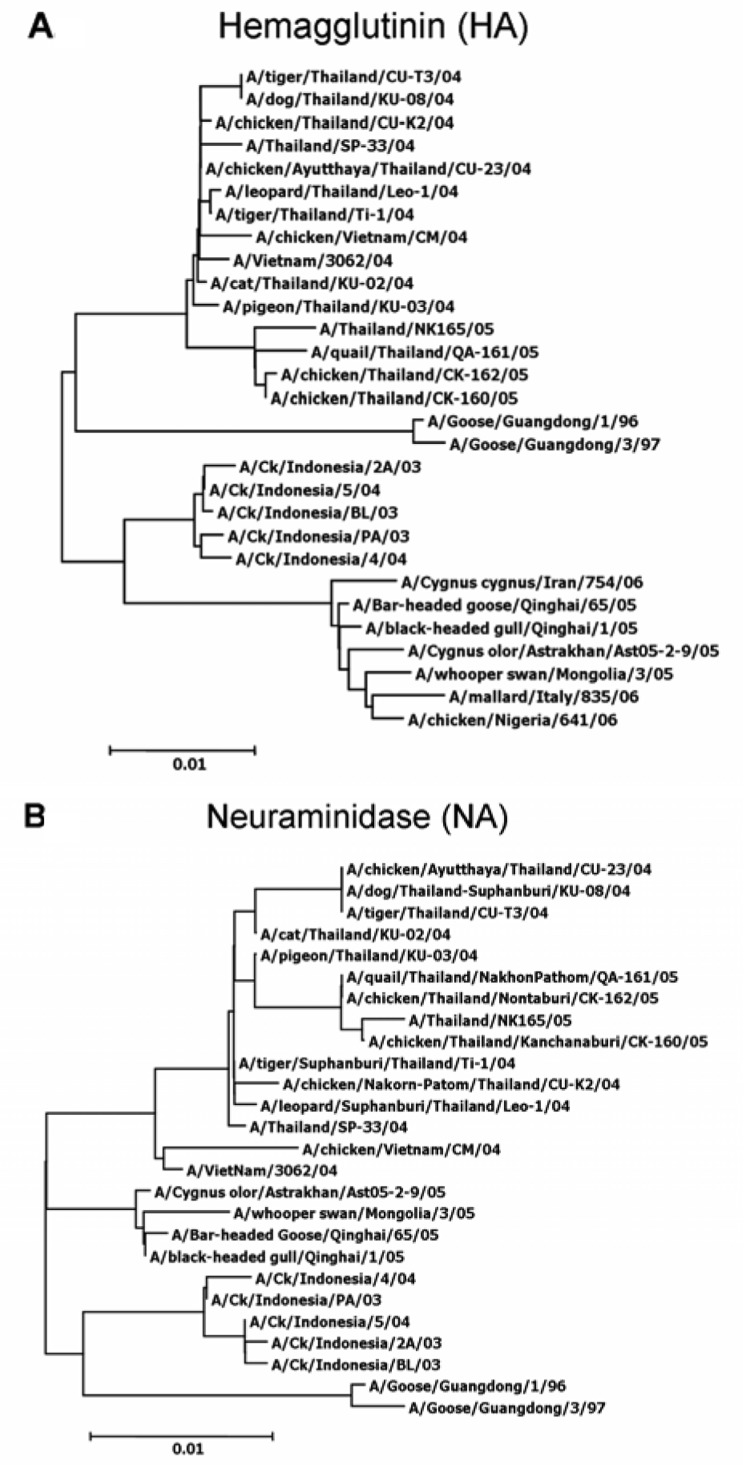
Phylogenetic analysis of the hemagglutinin (A) and neuraminidase (B) gene sequences of the H5N1 influenza virus isolated from a dog's lung (KU-08), compared with other HA and NA sequences stored in GenBank.

H5N1 viruses were isolated from the dog's lung tissue and designated A/Dog/Thailand/KU-08/04. Genetic analysis was used to characterize the dog's virus (KU-08), and the sequences were deposited at GenBank under accession number DQ530170-7. Sequencing and phylogenetic analysis of the hemaggluttinin (HA) and neuraminadase (NA) genes of the dog's virus showed that they were similar to those of H5N1 viruses isolated from tigers, chickens, ducks, and humans infected in Thailand during the same time that the dog was infected (Figure 2A and B). In addition, analysis of 6 other genes from KU-08 showed similar results (data not shown). Phylogenetic analysis clearly indicated that all the Thailand isolates were clustered with the Vietnam lineage, which groups separately from the Indonesia lineages and China (Qinghai), Europe, and Africa lineage. Genetic comparisons of the 8 genes analyzed from KU-08 to those of viruses isolated in Thailand from chickens (Jan 04, Jul 04, Oct 05), tigers (Jan 04, Oct 04), humans (Jan 04, Dec 05), cats (Jan 04), and geese (1996, Jun 05) are shown in the Table. The analysis showed that KU-08 was more closely related to the tiger isolate (CU-T3) obtained in Oct 2004, with higher percentages of nucleotide identity (100% identity for 5 genes: H5, N1, matrix [M], nonstructural [NS], polymerase basic protein 1 [PB1]) compared to any of the Thailand isolates obtained from early 2004 and late 2005.

**Table:** Genetic comparison of 8 gene segments of the H5N1 influenza virus isolated from the dog's lung (KU-08) to those of H5N1 isolates from Thailand*

KU-08: dog (Oct 04)†	Region of comparison	% nucleotide identity


GD-1: goose (1996)‡	CU-K2:chicken (Jan 04)§	SP-33: human (Jan 04)¶	Ti-1: tiger (Jan 04)#	KU-02: cat (Jan 04)**	CU-23: chicken (Jul 04)††	CU-T3: tiger (Oct 04)‡‡	CK-160: chicken (Oct 05)§§	NK-165: human (Dec 05)¶¶	QH-65: goose (Jun 05)##
HA	46–1623	96.0	97.1	99.0	99.6	99.6	99.6	100.0	99.2	98.9	96.8
NA	28–1296	91.6	99.3	99.3	99.5	99.5	99.9	100.0	98.5	98.8	97.2
M	1–801	96.1	99.5	99.6	99.4	99.9	99.9	100.0	99.4	99.3	98.1
NS	37–789	68.5	99.0	99.2	99.3	99.5	99.3	100.0	99.1	99.0	96.4
NP	58–1428	92.6	99.7	99.7	99.6	99.6	99.8	99.9	99.1	99.1	98.1
PA	28–2118	93.5	99.4	99.3	99.5	99.4	99.5	99.7	99.2	98.8	97.7
PB1	67–2229	93.4	99.6	99.8	99.8	99.8	99.9	100.0	99.1	98.8	97.9
PB2	88-2220	94.0	99.4	99.3	99.4	99.7	99.8	99.8	98.9	98.9	97.5

*HA, hemagluttinin; NA, neuraminadase; M, matrix; NS, nonstructural; NP,nucleoprotein; PA, polymerase A; PB, polymerase basic protein. †A/dog/Thailand-Suphanburi/KU-08/04 (DQ530170–7). ‡A/Goose/Guangdong/1/96 (AF144300–7). §A/chicken/Nakorn-Patom/Thailand/CU-K2/04 (AY590567- 8, AY590578–82, AY551934). ¶A/Thailand/SP-33/04 (AY555152–3, AY627893–8). #A/tiger/Suphanburi/Thailand/Ti-1/04 (AY646167–74). **A/cat/Thailand/KU-02/04 (DQ236077–84). ††A/chicken/Ayutthaya/Thailand/CU-23/04 (AY770991- AY7709918). ‡‡A/tiger/Thailand/CU-T3/04 (AY907672–3, AY842935–6, AY972547–50). §§A/chicken/Thailand/Kanchanaburi/CK-160/05 (DQ334757–64). ¶¶A/Thailand/NK165/05 (DQ372591–8). ##A/Bar-headed Goose/Qinghai/65/05 (DQ095622, DQ095642, DQ095662, DQ095682, DQ095702, DQ095722, DQ095742, DQ095762).

This finding indicates that the dog's H5N1 infection resulted from the virus circulating during the second wave of H5N1 outbreaks that occurred in Thailand during mid-2004. The HA gene of KU-08 contained multiple basic amino acid insertions at the HA cleavage site (SPQRERRRKKRR), similar to those found at the HA cleavage site for other viruses characterized from Thailand during this time. However, the isolates from the third wave of AI outbreaks that occurred in Thailand in 2005 contained 1 basic amino acid (aa) change at the HA cleavage site (SPQREKRRKKRR) (*7*). Moreover, the viruses isolated from China (A/Bar-headed goose/Qinghai/65/05, A/Black-headed gull/Qinghai/1/05) (*8*), Iran (A/Cygnus cygnus/Iran/754/06), Russia (A/Cygnusolor/Astrakhan/Ast05–2-9/05), and Nigeria (A/chicken/Nigeria/641/06) displayed a different amino acid at the HA cleavage site (SPQGERRRKKRR). The receptor-binding site of the dog isolate still exhibited avian characteristics in that it contained glutamine (Q) and glycine (G) at positions 222 and 224 of the HA gene (Q222–G224). The NA gene of KU-08 also had 20 aa deletions at positions 49–68 and contained histidine (H) amino acid at position 274, indicating the absence of an oseltamivir-resistant residue. The NS gene of the KU-08 isolate contained a 5-aa deletion at positions 79–83, and the M gene of the KU-08 isolate displayed an amantadine-resistant amino acid (N31; asparagine). In summary, the viruses from the dog were similar to the H5N1 viruses isolated in Thailand in 2004 and to the Vietnam lineage which had been identified as genotype Z (*9*). A single amino acid substitution at position 627 of the PB2 gene (glutamic acid [E] to lysine [K]) was observed in KU-08 and had previously been observed in human, tiger, and cat isolates from Thailand as well as the viruses from China (Qinghai). The presence of lysine (K) may relate to more efficiency of viral replication in mammal species (*10*). On the other hand, in pigeon isolates from Thailand (KU-03), the PB2–627 aa residue remained unchanged (E; glutamic acid).

This study is the first report of H5N1-related systemic disease in a domestic dog infected during the second wave of outbreaks in Thailand that occurred during October 2004. The most plausible route of the dog's infection was ingestion of infected duck carcasses. Previous studies have shown that avian viruses preferentially recognize a-2,3 linkage (SAa2,3Gal) and bind to type II alveolar cells, which are abundant in the lower respiratory tract of mammals (*11**,**12*); these findings support our observations of severe pneumonia with lung edema in the infected dog (Figure 1). Characterization of the H5N1 isolates from the dog showed identical properties to the H5N1 isolates from the Thai epidemic. Moreover, genetic comparison indicated that the dog isolate was similar to the H5N1 viruses recovered from a tiger (CU-T3) in Thailand during the mid-2004 epidemic.

## Conclusion

Our results demonstrate that, as has previously been shown for cats, dogs are at risk for H5N1 infection. Despite the low probability of H5N1 infection in domestic animals, the possibility of humans acquiring H5N1 infection from direct contact with infected cats and dogs warrants concern and highlights the need for monitoring domestic animals during H5N1 outbreaks in the future.
